# Facile Synthesis and Optical Properties of Small Selenium Nanocrystals and Nanorods

**DOI:** 10.1186/s11671-017-2165-y

**Published:** 2017-06-12

**Authors:** Fengrui Jiang, Weiquan Cai, Guolong Tan

**Affiliations:** 1 State Key Laboratory of Advanced Technology for Materials Synthesis and Processing, Wuhan, 430070 China; 2 School of Chemistry, Chemical Engineering and Life Sciences, Wuhan University of Technology, Wuhan, 430070 China

**Keywords:** Se nanoparticles, Glycerin, Optical properties, Microstructure

## Abstract

Selenium is an important element for human’s health, small size is very helpful for Se nanoparticles to be absorbed by human's body. Here, we present a facile approach to fabrication of small selenium nanoparticles (Nano-Se) as well as nanorods by dissolving sodium selenite (Na_2_SeO_3_) in glycerin and using glucose as the reduction agent. The as-prepared selenium nanoparticles have been characterized by X-ray diffraction (XRD), UV-Vis absorption spectroscopy and high resolution transmission electron microscope (HRTEM). The morphology of small Se nanoparticles and nanorods have been demonstrated in the TEM images. A small amount of 3-mercaptoproprionic acid (MPA) and glycerin play a key role on controlling the particle size and stabilize the dispersion of Nano-Se in the glycerin solution. In this way, we obtained very small and uniform Se nanoparticles; whose size ranges from 2 to 6 nm. This dimension is much smaller than the best value (>20 nm) ever reported in the literatures. Strong quantum confinement effect has been observed upon the size-dependent optical spectrum of these Se nanoparticles.

## Background

Nanomaterials have become the focus of many research areas due to their unique physical and chemical properties. Various nanoparticles, such as titanium oxide, silver, gold, and cadmium selenide nanoparticles, are already being used in catalysis, stain-resistant clothing, sunscreens, cosmetics, and electronics [1–3]. Pure selenium, as well as selenium containing nano-materials, has excellent photoelectrical characteristics, semiconductor properties, and high biological activity [3]. Selenium nanomaterials with 1D structure are one of the key materials by virtue of their broad applications in optoelectronics devices such as rectifiers, photocopiers, photographic exposure meters, xerography, and solar cells due to its high photoconductivity [4–6].

As an important inorganic material, selenium also has attracted a great deal of attention for owing good semiconducting behavior with band gap value of 1.6 eV [7, 8]. What is more important, nano selenium play an important role on the biology and medicine, in virtue of their excellent biological activities and low toxicity [9–14], which makes it species being capable of selectively killing cancer cells constitutes an urgent priority [15, 16]. Selenium is an essential trace element, which is present in most foods, for human health. Selenium is present in foods mainly as the amino acids selenomethionine and selenocysteine. Selenium compounds are anti-oxidants that delete free radicals in vitro and improve the activity of the seleno-enzyme, glutathione peroxidase, which can prevent free radicals from damaging cells and tissues in vivo [17–19]. Recently, selenium nanoparticles have been used as additives in growth of corn and cereals as well as in vitamins replacing organic selenium compound to replenish the essential trace selenium in human’s body.

Over the past few years, Se nanoparticles, nanorods, nanowires and nanotubes [20–24] have been generated by many strategies [5, 24, 25]. For example, the hydrothermal method reported by Rao’s group [26], the carbothermal chemical vapor deposition route suggested by Zhang’s group [27], all required relatively rigorous reaction conditions. The chemical methods based on solution-phase procedures seem to provide an excellent route to fabricate Nano-Se. However, the size of these Se nanoparticles prepared by the above methods is very big (>20 nm), some of them are larger than 100 nm, which could somehow reduce the absorbance efficiency of selenium in human’s body. Obviously, developing effective and environment friendly routes to fabricate large quantities of small Se nanoparticles with small particle size (<10 nm) is still facing challenges, but it is essential for health care application.

Herein, we present a controllable and rapid approach to fabrication of small Se nanoparticles with a size less than 6 nm by using glucose as the reduction agent and glycerin as the stabilization agent. In comparison with the previous studies, this method is green and environment friendly since glycerin and glucose are compatible with the cells in the human bodies. Smaller size would improve the absorbance efficiency of selenium nanoparticles in a human’s body and therefore would be widely used in supplying the trace selenium element in foods, vitamins and other medicines.

## Methods

Na_2_SeO_3_ powder, glycerin glucose powder, ethanol, 3- mercaptoproprionic acid (MPA) (99%, Alfa Aesar) were all used without additional purification. Firstly, a stock precursor solution of Na_2_SeO_3_ was prepared by dissolving 0.023 g Na_2_SeO_3_ powder in a mixture of 20 mL distilled water and 2 mL ethanol, then 18mL glycerin was added into the above solution. The reduction agent was prepared by dissolving 1.0076 g glucose powder in a mixture of 20 mL distilled water and 1 mL MPA. The precursor solution of Na_2_SeO_3_ was heated to 60 °C, then the reduction agent of glucose was injected into the precursor solution. Afterwards, the mixture solution was gradually heated to 120 °C for 3 min, the dispersion solution became dark red from limpidity, indicating the formation of Se nanoparticles through the following reduction reaction:

$$ \begin{array}{l} N{a}_2 Se{O}_3\to N{a}_2 O+ Se{O}_2\\ {} Se{O}_2+2{C}_5{H}_6{(OH)}_5 CHO\to Se\downarrow +2{C}_5{H}_6{(OH)}_5 COOH\end{array} $$

In this way, Se nanoparticles have been fabricated, the residue solvent was composed of Na_2_O, gluconic acid, MPA and water. Excess glucose was applied so as to ensure the reduction reaction was fully completed. At different temperature steps, small amount (7 mL) of dispersion solution with Se nanoparticles was suctioned into a small glass bottle for optical and TEM measurement. Small selenium nanoparticles dispersing in the glycerin solution were thus obtained. The dispersion solution was aged for 45 days and then washed several times with distilled water. The selenium nanoparticles grew gradually into nanorods during the aging process.

The as-prepared products were characterized by using various methods. The sample for the X-ray diffraction (XRD) was prepared by centrifugation of the dispersion solution with selenium nanoparticles at 12,000 rps/s for 30 min, and then powders were heated at 400 °C for 1 h to fully crystallize the nanocrystals. The microstructure features of as-prepared Se nanoparticles were measured by a JEOL 2100F high resolution transmission electronic microscopy (HRTEM). UV–vis optical spectra of the dispersion solution with Se nanoparticles or nanorods have been collected by a Phenix 1900PC UV-Vis-NIR Spectroscopy.

## Results and Discussions

### Structure Identification of Se Nanoparticles

**Fig. 1:**
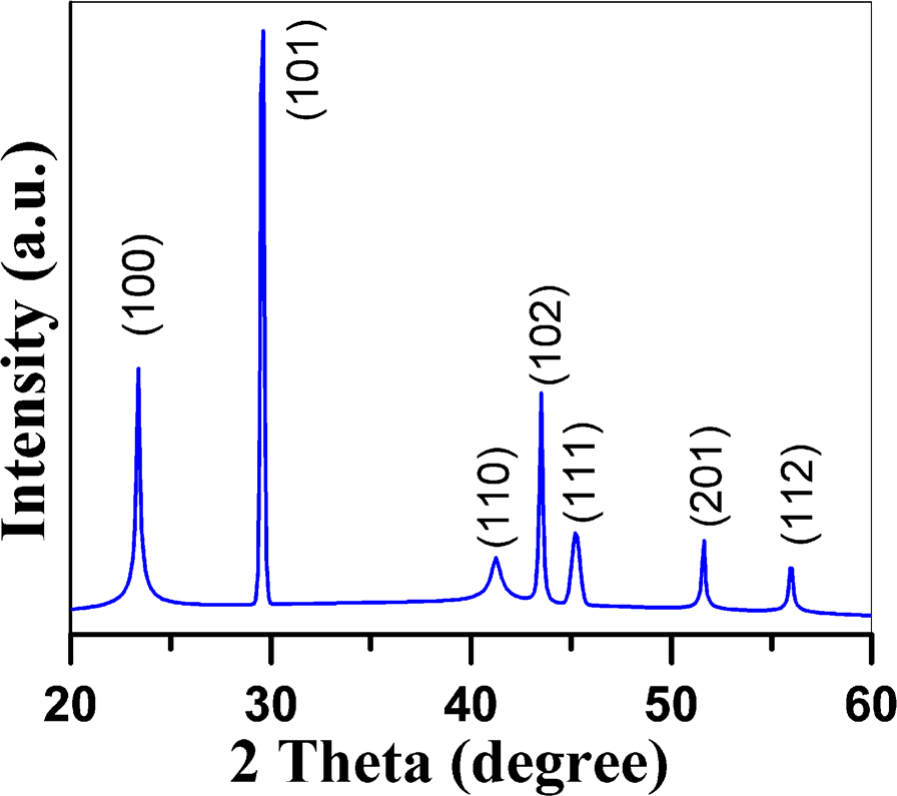
XRD diffraction pattern of Se nanoparticles being calcined at 400 °C for 1 h

For the XRD measurement of Se nanparticles, some of the dispersion solution was purged by water and alcohol for three times following the centrifugation process. Se nanoparticles lost its activity and became dark when they are separated from the dispersion solution and exposed to air. In order to obtain information about the structure and the size of Se nanoparticles, two types of samples were prepared for XRD and TEM measurement, respectively. The first one was separated from the freshly synthesized Se colloid suspension which was heated at 80 °C for 3 min and the second one from nanopowders, which were calcined from the centrifuged colloid dispersion solutions at 400 °C for 1 h. The freshly prepared Se nanoparticles are amorphous (a-Se), while the other Se nanoparticles being annealed at 400 °C are well crystallized. The XRD diffraction peaks (Fig. 1) are indexed to (100), (101), (110), (102), (111), 200), (201), and (003) lattice planes of hexagonal Se, being in good agreement with the characteristic peaks in the standard card (PDF 65-1876) [24, 28]. Careful analysis of the above XRD pattern revealed that the Se nanoparticles have been crystallized in pure trigonal phase. Lattice constants are determined to be *a* = 0.437 nm and *b* = 0.496 nm from this XRD pattern, being consistent with those values reported in the literatures (*a* = 0.436 nm, *b* = 0.495 nm) [29].

### Optical Properties of Se Nanoparticles

**Fig. 2:**
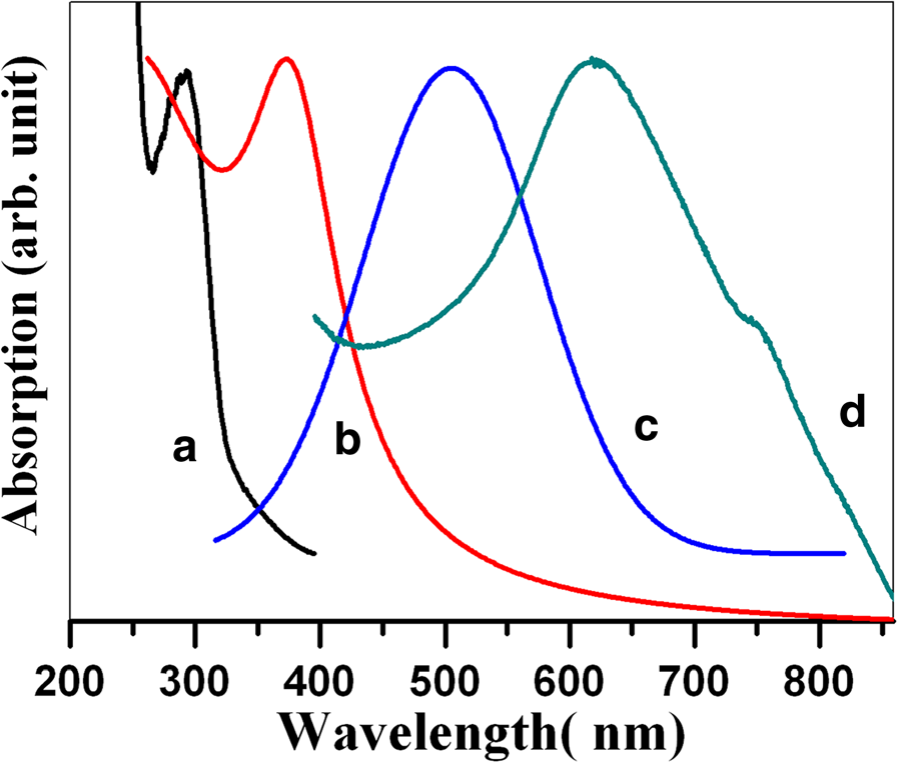
UV–visible optical spectra of Nano-Se being prepared at different temperatures: (**a**) 60 °C, (**b**) 80 °C, (**c**) 100 °C for 30 min; (**d**) 120 °C plus aging for 45 days

The whole experiment process was going along with the color change. Firstly, when the MPA and the glucose solution were just added into the Na_2_SeO_3_ precursor solution, the mixture became pellucid; lately, when the temperature increases from 60 to 120 °C, the color of the dispersion solution changed from pale yellow to bright orange, following by blood orange and finally to deep red. Such a color change of Se nanoparticles dispersion could be more clearly manifested by the UV–visible absorption spectra in Fig. 2, which displayed the optical spectra of the specimens being prepared at the temperatures of 60, 80, 100, and 120 °C. In order to prevent the loss of activity of fresh Se nanoparticles, the optical measurement was carried out upon the freshly prepared dispersion solution. The remaining solvent (gluconic acid, MPA, glycerin) are all transparent colorless solution and would show a absorption peak at around 240 nm, but would not present any absorption peaks within the visible wavelength region. Therefore, the absorption peaks in Fig. 2 are all contributed from Se nanoparticles. It can be seen that the original absorption peak of Se nanocrystals locates at 292 nm (a), which shifts to 371 nm (b) when the reaction temperature rises to 80 °C and further red shifts to 504 nm (c) and 618 nm (d) when the nano-Se suspension was heated up to 100 and 120 °C, respectively. These multiple absorbance peaks of Nano-Se are coupled with the reaction temperature. The higher is the temperature, the larger is the particle size. The red-shifted optical spectra of Se nanoparticles with particles size was actually confined by the quantum size effect (Fig. 2).

Selenium is a typical direct semiconductor with a band gap energy of 1.6 eV (775 nm). When particle size is smaller than its Bohr excitation radius, the band gap will be enlarged due to the quantum confinement effect. Therefore, the optical absorption spectrum demonstrates a big blue shift of the band gap energy for Se nanocrystals in comparison with its bulk counterpart. The absorption peak shifts from 775 nm (bulk Se) to 292 nm for Se nanoparticles (being fabricated at 60 °C). When the reaction temperature rises to 80 °C, the absorption peak for the Se nanoparticles red shifts to 371 nm and further to 504 nm when the reaction temperature increases to 80 and 100 °C, respectively. Finally the absorption peak moves to 618 nm when the suspension of Se nanoparticles was heat treated at 120 °C for 30 min and aged for another 45 days. The total shift of the band gap energies for Se nanoparticles values 483 nm (0.39 eV) in comparison with that of bulk counterpart. The band gap energy of Se nanocrystals decreases with the particle size, which changes with the reaction temperature. The bigger is the particles size, the smaller is the band gap energy. The origin of the shift of the absorption peaks with temperature is induced by the well-known quantum confinement effect, which leads to the color change of the Se nanoparticles suspension.

### Microstructure of Se Nanoparticles

**Fig. 3:**
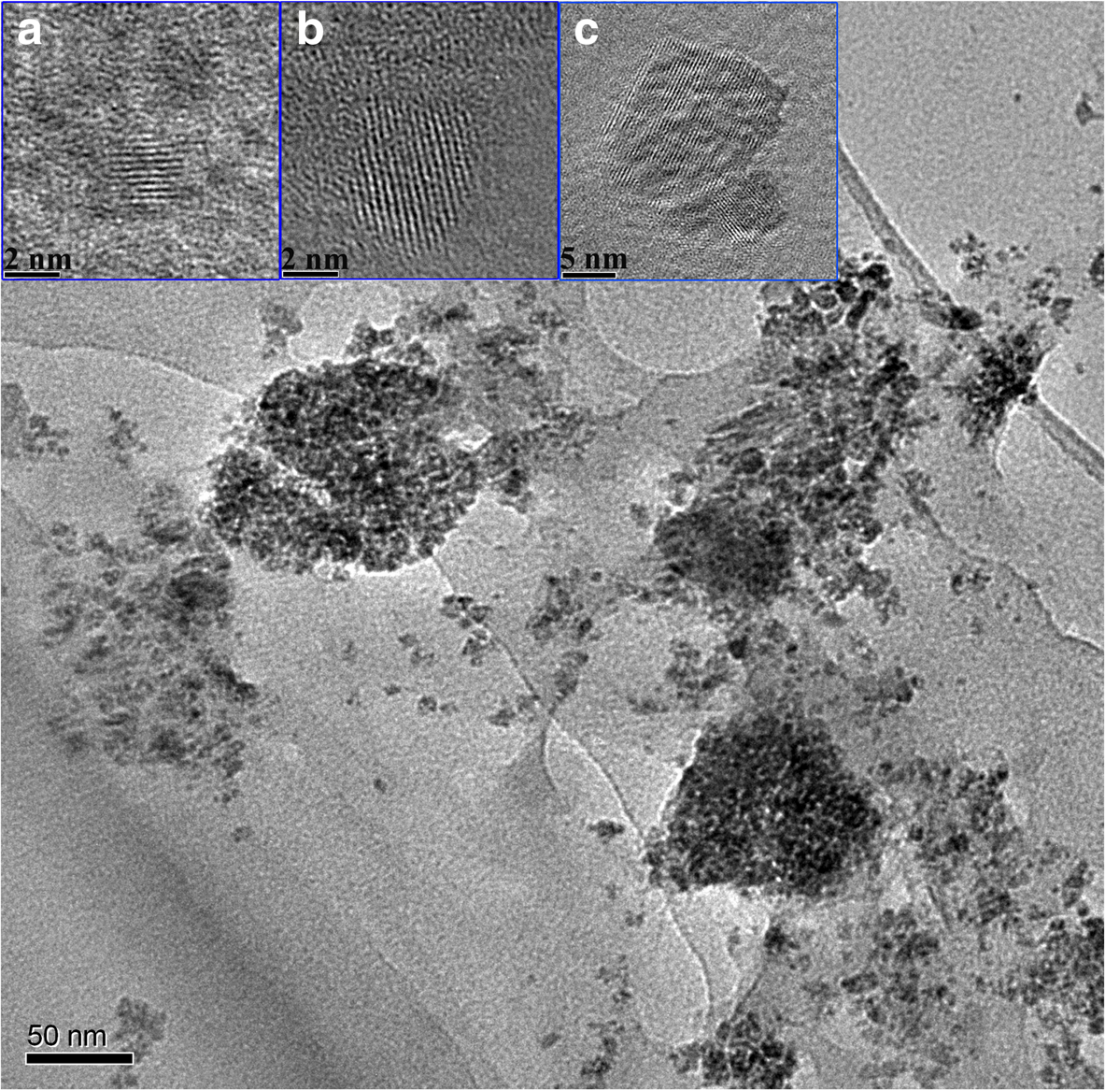
TEM and HRTEM images of water soluble Se nanoparticles being collected from the freshly synthesized Se suspension

The microstructure and morphology of the as-prepared Se nanoparticles are exhibited in Fig. 3 which shows TEM images of as-prepared Se nanoparticles using MPA as the stabilizing agent at the pH value of 11. The particle size ranges from 2~10 nm; averaging at 4.8nm. This image exhibits lots of small Se nanoparticle with a little aggregation, The insets are three HRTEM images of three individual Se nanoparticles, whose lattice fringes are clearly seen. Image (a) shows a very small Se nanoparticles with size less than 3 nm, image (b) demonstrates one Se nanoparticle in size of 5 nm and image (c) a little large particle with size of around 10 nm. The lattice fringes are clearly seen in these nanocrystals, most of the fringes are assigned to {101} lattice planes in hexagonal structure. The lattice spacing for these one dimensional fringes has been determined to be 2.978 Å from the Fast Fourier Transformation of the HRTEM images in reciprocal space, the value matches lattice spacing of {101} lattice planes. It is hard to determine the orientation of these nanoparticles due to the appearance of only one-dimensional lattice fringes. The HRTEM images of individual nanoparticles further confirm the hexagonal structure of the as-prepared Se nanoparticles, being consistent with the XRD results. The smallest Se nanoparticles observed in our HRTEM images is about 2 nm in diameter, as being seen in Fig. 3d. It can be seen from the HRTEM images that these nanoparticles are well crystallized with rare defects. Dislocations stacking faults and twins are not observed in these particles, indicating that these kinds of water soluble Se nanoparticles are almost defect free.

Until now, fabrication of small Se nanoparticles smaller than 10 nm was proved to be very difficult. The size of Se nanoparticles was reported to be larger than 20 nm [30], some of them are even larger than 50 nm [31–33]. It seems to be very hard to control the rapid growth of Se nanoparticles with reaction time in traditional chemical process.

**Fig. 4:**
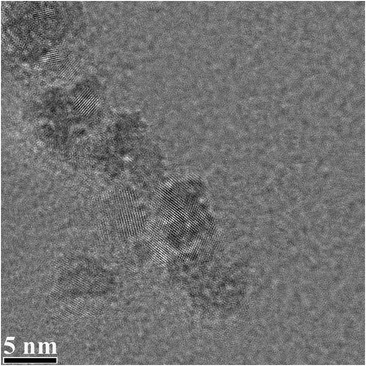
High resolution transmission electron microscopy (HRTEM) images of water-soluble Se nanoparticles

In our case, the size of Se nanoparticles are well controllable. These Se nanoparticles exhibit homogeneous size distribution, which ranges from 2 to 6 nm with occasionally occurrence of big particles over 6 nm, as being shown in Fig. 4. Actually, the HRTEM images in Fig. 4 were taken directly from the suspension solution of Se nanoparticles after the specimen was aging for 3 weeks, indicating that Se nanoparticles are stable in the glycerin containing solution.

**Fig. 5:**
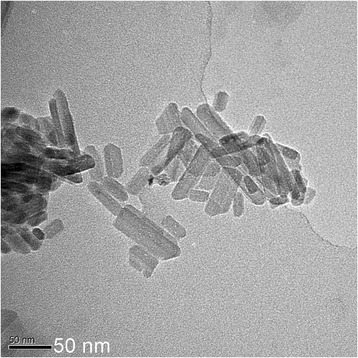
TEM image for Se nanorods, which was growing up from the Se nanoparticles aging for 9 days after glycerin was removed away

**Fig. 6:**
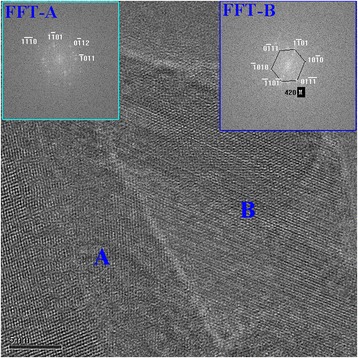
HRTEM image for Se nanorods in hexagonal structure after the particle specimen was cleaned up and aging for 3 months

**Fig. 7:**
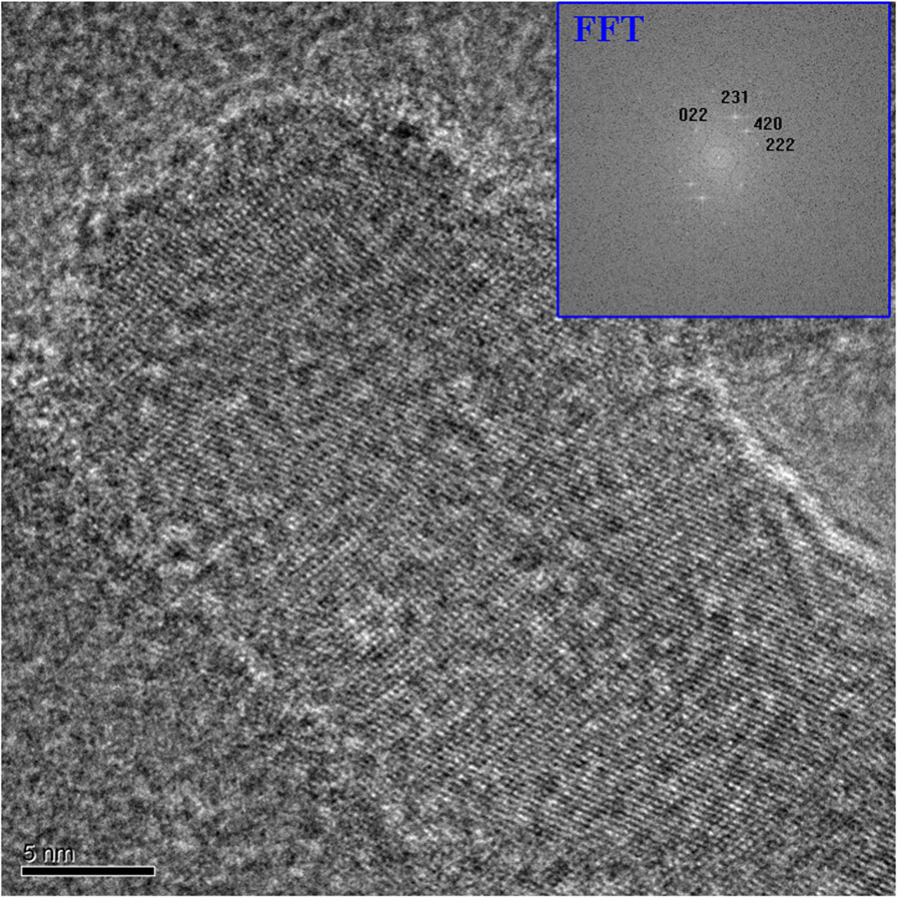
HRTEM image for Se nanorod in monoclinic structure after the particle specimen was cleaned up and aging for 3 months

However, when these Se nanoparticles were cleaned up for several times by water, the dispersion solution changed to black color due to the growth of the particle size, which was larger than 50 nm. Some of the particles even grew up to nanorods with length of several hundreds of nanometers. Once glycerin was removed away from the surface of Se nanoparticles after cleaning process and let the particles age in air for more than 3 months, Se nanoparticles would lose activities and then grew up rapidly into nanorods along [022] or [110] direction (Fig. 5). The morphology of these nanorods developing from aging of small Se nanoparticles are demonstrated in Fig. 5. This kind of Se nanorods are rarely reported in the literatures [28–30]. The HRTEM image as well as Fourier transformation of the images for these Se nanorods are shown in Figs. 6 and 7, which display the hexagonal and monoclinic structure respectively. There are two nanorods in Fig. 6, both in hexagonal structure. Rod A is in orientation of $\left[01\overline{1}1\right]$, while rod B in orientation of $\left[1\overline{2}1\overline{3}\right]$. The growth direction of rod A and B are (110) and (001), respectively. However, the Se nanorod in Fig. 7 is in monoclinic structure, which grows in the direction of (022). Therefore, the aging Se nanoparticles all transformed into nanorods, which are actually composed of two crystal structures, one is hexagonal structure and the other one is monoclinic structure. When Se nanoparticles were dispersed in glycerin solution, small Se nanoparticles were pretty stable and they would not grow up into big particles or nanorods with aging time.Glycerin plays a key role on suppressing the growth of Se nanoparticles and could keep Se nanoparticles in high activity in the solution. After the particles were cleaned up, the glycerin was removed away, Se nanoparticles lost its activity and grew rapidly into nanorods alone a certain direction. Meanwhile, glycerin is a kind of biology compatible organic compound; Se nanoparticles being stabilized by such a biology friendly agent would somehow have potential application in health products to provide Se sources for human body.

## Conclusions

A new facile and green method for the synthesis of small uniform Se nanoparticles has been presented. In this method, glucose was used to reduce Na_2_SeO_3_ to fabricate Se nanoparticles, glycerin was utilized as stabilizing agent to suppress the abnormal growth of Se nanoparticles. Here, glycerin plays a key role on controlling the size of selenium nanoparticles and its stability in the solution. By this way, water-soluble Se nanoparticles with size ranging from 2 to 6 nm have been obtained. These Se nanoparticles demonstrates strong quantum confinement effect, the optical absorption spectrum demonstrates a large blue shift of the band gap energy for the Se nanocrystals in comparison with its bulk counterpart. The band gap energy for Se nanoparticles blue shifts from 775 nm (bulk) to 292 nm. It is a green and environment-friendly synthesis process, what is more important; the size of Se nanoparticles could reach as small as 2 nm with narrow size distribution. These Se nanoparticles in glycerin solution are biological compatible with potential application in medicine field.

## Abbreviations

1D: 1 Dimension
eV: Electron voltage
HRTEM: High resolution transmission electron microscopy
MPA: 3- mercaptoproprionic acid
nm: Nanometer
TEM: Transmission electron microscopy
XRD: X-ray diffraction
